# Endoscopic submucosal dissection for treatment of esophageal leukoplakia reveals hidden histopathology

**DOI:** 10.1055/a-2271-5679

**Published:** 2024-03-20

**Authors:** Moritz Meiborg, Tobias Blasberg, Marie Weber, Johannes Richl, Dirk Freitag, Edris Wedi

**Affiliations:** 1 Department of Gastroenterology, Gastrointestinal Oncology and Interventional Endoscopy, Sana Klinikum Offenbach, Offenbach, Germany; 2 Department of Gastroenterology and Endocrinology, MVZ am Mathilden-Hospital Büdingen, Büdingen, Germany

A 59-year-old woman with a medical history of severe reflux esophagitis, chronic obstructive pulmonary disease, and tobacco and alcohol dependence was referred to our clinic for an assessment of progressive hyperkeratotic lesions in the mid-section of the esophagus, which were incidentally found on routine endoscopic surveillance. Prior to the initial presentation at our clinic, the patient had undergone endoscopic monitoring for a duration of 2.5 years. With the exception of sporadically occurring reflux symptoms, the patient remained asymptomatic. There was no indication of B-symptoms.

At the initial esophagogastroduodenoscopy, distinct white plaques with a cobblestone appearance were detected in the mid-section of the esophagus ( Fig. 1 ). The lesions extended semi-circumferentially from approximately 28–30 cm and revealed no staining on Lugol’s iodine chromoendoscopy ( Fig. 2 ). Histopathological examination confirmed esophageal epidermoid metaplasia, consistent with esophageal leukoplakia. No dysplastic cells were detected, and Gomori methenamine–silver nitrate stain for fungal organisms showed negative results ( Fig. 3 ).

**Fig. 1:**
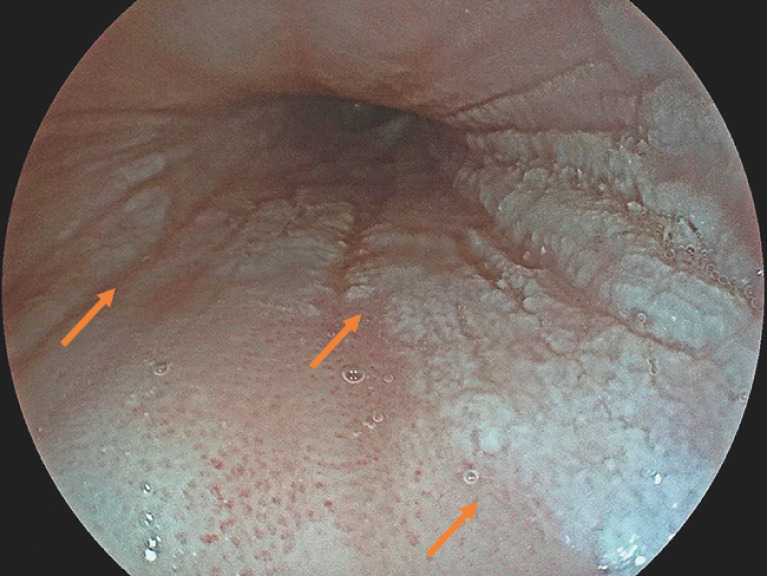
Esophageal leukoplakia. White, well-demarked lesions (arrows) with cobblestone appearance in the mid-section of the esophagus.

**Fig. 2:**
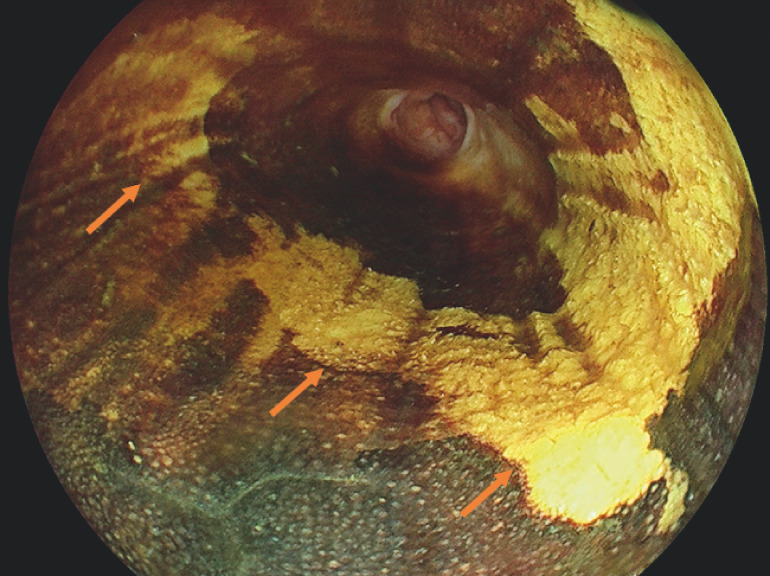
Lugol’s iodine chromoendoscopy. The suspected lesions showed absence of staining.

**Fig. 3:**
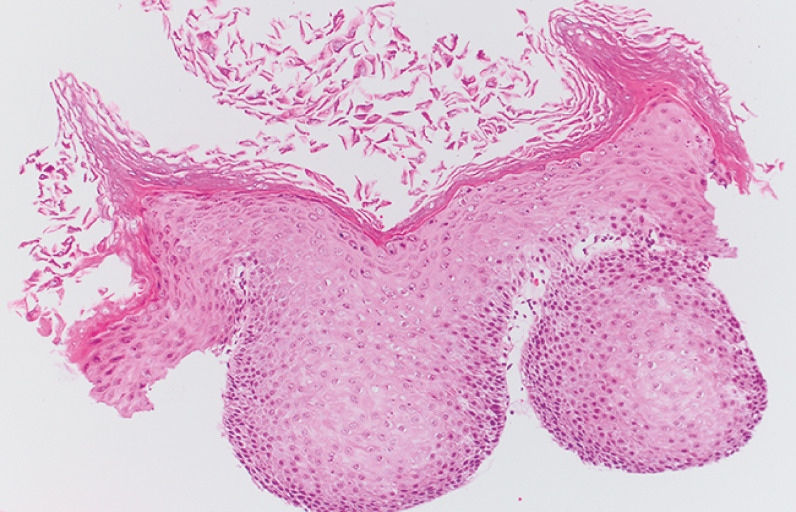
Histopathological examination I. Esophageal epidermoid metaplasia, consistent with esophageal leukoplakia. No dysplastic cells were detected. Gomori methenamine–silver nitrate stain for fungal organisms showed negative results.

According to current research, esophageal leukoplakia can occur adjacent to high grade squamous cell carcinoma and is perceived as a precursor lesion 1 2 . Thus, contrast-enhanced computed tomography scans of the chest and abdomen were performed, demonstrating no signs of distant metastases and pathological lymph nodes. Subsequent evaluation with a radial echoendoscope (EG-580 UR; Fujifilm, Tokyo, Japan) showed lesions limited to the mucosa and therefore amenable to endoscopic therapy. Due to the rarity of the disease, guidelines for surveillance and treatment have not yet been established. The approaches predominantly described in the literature include either frequent endoscopic surveillance or treatment via radiofrequency ablation or argon plasma coagulation 3 . Regarding the growth pattern, extent of the lesions, and potential for malignant transformation, endoscopic submucosal dissection (ESD) was suggested to ensure a complete resection.

Underwater tubular ESD was accomplished using the FlushKnife N-S (DK2623J-N15; Fujifilm) with forced coagulation mode (Endo Cut Q, effect setting 2, cutting duration setting 1, cutting interval setting 1; Erbe Elektromedizin, Tübingen, Germany) ( Video 1 ). No adverse events occurred. The final histological examination revealed a high grade verrucous squamous cell carcinoma (pT1a, m1, G1), with en bloc R0 resection ( Fig. 4 ). During the 4-month follow-up, no evidence of recurrence or post-ESD esophageal stricture was observed ( Fig. 5 ).

Endoscopic submucosal dissection for treatment of esophageal leukoplakia: a novel approach for treatment in cases with marked esophageal involvement and a high risk of recurrence.Video 1

**Fig. 4:**
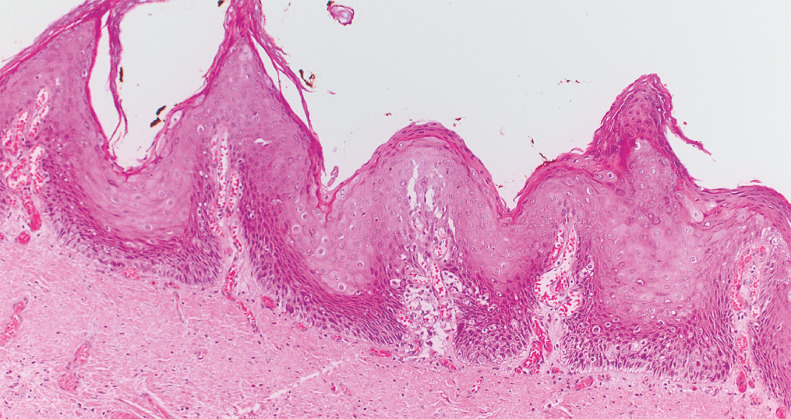
Histopathological examination II. High grade verrucous squamous cell carcinoma (pT1a, m1, G1), with en bloc R0 resection.

**Fig. 5:**
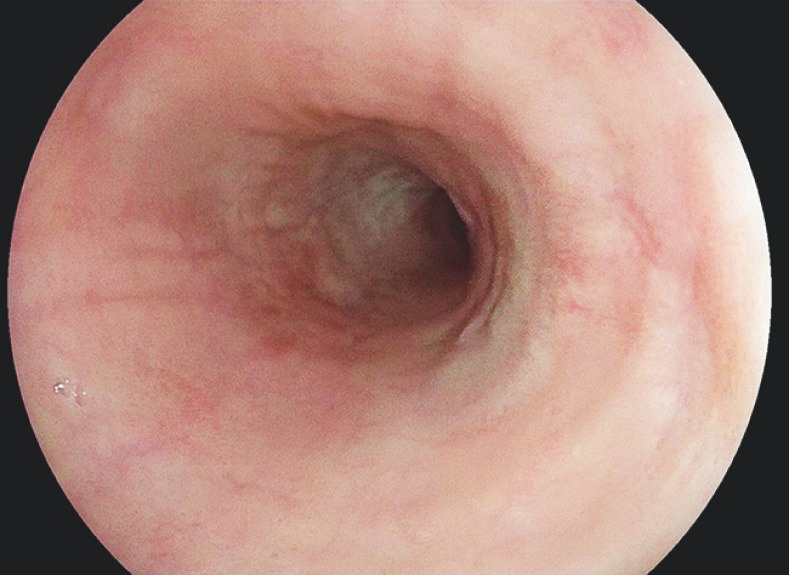
At the 4-month endoscopic follow-up, there was no evidence of recurrence or post-endoscopic submucosal dissection esophageal stricture.

In conclusion, ESD can be considered as an effective and safe therapy option for esophageal leukoplakia in cases with marked esophageal involvement and a high risk of recurrence.

Endoscopy_UCTN_Code_CCL_1AB_2AC_3AB

## Notes

Endoscopy E-Videos https://eref.thieme.de/e-videos
*E-Videos* is an open access online section of the journal *Endoscopy* , reporting on interesting cases and new techniques in gastroenterological endoscopy. All papers include a high-quality video and are published with a Creative Commons CC-BY license. Endoscopy E-Videos qualify for HINARI discounts and waivers and eligibility is automatically checked during the submission process. We grant 100% waivers to articles whose corresponding authors are based in Group A countries and 50% waivers to those who are based in Group B countries as classified by Research4Life (see: https://www.research4life.org/access/eligibility/ ). This section has its own submission website at https://mc.manuscriptcentral.com/e-videos .
